# Efficacy and tolerability of folate-aminopterin therapy in a rat focal model of multiple sclerosis

**DOI:** 10.1186/s12974-021-02073-7

**Published:** 2021-01-20

**Authors:** Petri Elo, Xiang-Guo Li, Heidi Liljenbäck, Maria Gardberg, Olli Moisio, Maxwell Miner, Jenni Virta, Antti Saraste, Madduri Srinivasarao, Michael Pugh, Philip S. Low, Juhani Knuuti, Sirpa Jalkanen, Laura Airas, Yingjuan June Lu, Anne Roivainen

**Affiliations:** 1 Turku PET Centre, University of Turku, Turku, Finland; 2 Turku PET Centre, Åbo Akademi University, Turku, Finland; 3 Turku Center for Disease Modeling, University of Turku, Turku, Finland; 4 Department of Pathology, Turku University Hospital and Institute of Biomedicine, University of Turku, Turku, Finland; 5 Turku PET Centre, Turku University Hospital, Turku, Finland; 6 Department of Chemistry, Purdue University, West Lafayette, IN USA; 7 Endocyte, Inc., now part of Novartis Institutes for Biomedical Research, West Lafayette, IN USA; 8 MediCity Research Laboratory, University of Turku, Turku, Finland; 9 Department of Neurology, Turku University Hospital, Turku, Finland

**Keywords:** Aminopterin, Folate receptor, Experimental autoimmune encephalomyelitis, Inflammation, Macrophages, Multiple sclerosis, Positron emission tomography

## Abstract

**Background:**

Activated macrophages in the experimental model of multiple sclerosis (MS) express folate receptor-β (FR-β), representing a promising target for the treatment of MS. Here, we both evaluated the efficacy of a novel folate-aminopterin construct (EC2319) in a rat focal model of multiple sclerosis (MS) and investigated the utility of ^68^Ga-labeled 1,4,7-triazacyclononane-1,4,7-triacetic acid-conjugated folate (^68^Ga-FOL) for assessing inflammatory lesions. In addition, we investigated whether FR-β is expressed in the brain of patients with MS.

**Methods:**

Focal delayed-type hypersensitivity experimental autoimmune encephalomyelitis (*f*DTH-EAE) was induced in 40 Lewis rats; 20 healthy Lewis rats were used as controls. Rats were divided into six groups according to the duration of disease (control, acute, or chronic) and intervention (vehicle versus EC2319). ^68^Ga-FOL analyses, histology, and immunofluorescence of the brain were performed to evaluate the efficacy of subcutaneously administered EC2319 on lesion development. Immunofluorescence was used to assess FR-β expression in postmortem brain samples from 5 patients with MS and 5 healthy controls.

**Results:**

Immunofluorescence and histological analyses revealed significant reductions in FR-β expression (*P* < 0.05) and lesion size (*P* < 0.01), as well as improved inducible nitric oxide synthase/mannose receptor C type 1 ratios (*P* < 0.01) in macrophages and microglia during the chronic but not acute phase of *f*DTH-EAE in EC2319-treated rats. The uptake of IV-injected ^68^Ga-FOL in the brain was low and did not differ between the groups, but the in vitro binding of ^68^Ga-FOL was significantly lower in EC2319-treated rats (*P* < 0.01). FR-β positivity was observed in chronically active lesions and in normal-appearing white matter in MS brain samples.

**Conclusions:**

EC2319 was well tolerated and attenuated inflammation and lesion development in a rat model of a chronic progressive form of MS. Human MS patients have FR-β-positive cells in chronically active plaques, which suggests that these results may have translational relevance.

## Background

Most patients with a progressive form of multiple sclerosis (MS) respond minimally to current disease-modifying agents [1, 2], and many patients discontinue treatment because of undesirable side effects [3]. Thus, novel treatments are urgently needed. As activated macrophages in animals with experimental autoimmune encephalomyelitis (EAE) [4, 5], a model of MS, express folate receptor-β (FR-β), this represents an auspicious target for treating MS.

FRs overexpressed on cancer cells and activated macrophages can be targeted with a novel folate-aminopterin derivative, EC2319 [6]. Folate-aminopterin therapy reduces inflammation in acute myelin basic protein-induced EAE, but its effects on chronic forms are not known. There is evidence that the aminopterin conjugate exerts antineoplastic and immunomodulatory effects once it is internalized via FR binding, thereby inhibiting dihydrofolate reductase and possibly suppressing immune cell proliferation and cytokine recruitment. In association with central nervous system (CNS) inflammation, a functional FR-β has been shown to be present in a subpopulation of infiltrating inflammatory macrophages identified as the cluster of differentiation 68 (CD68)-positive subset of high major histocompatibility complex (MHC) II-expressing and high CD11b-expressing cells, and folate-aminopterin therapy has significantly reduced CD68-positive macrophages, inflammation, and demyelination [4]. Indeed, aminopterin derivatives have gained interest because of their superior anti-inflammatory effects and improved safety profile compared to methotrexate [7–9]. Here, we investigated the efficacy of subcutaneously administered EC2319 on lesion development during acute and chronic EAE in rats. In addition to immunofluorescence and histological analyses, we used FR-targeted PET, a promising approach for imaging activated macrophages under inflammatory conditions [5, 10–14]. For this, we used a ^68^Ga-labeled 1,4,7-triazacyclononane-1,4,7-triacetic acid-conjugated folate (^68^Ga-FOL). Furthermore, we assessed the expression of FR-β in postmortem brain samples of MS patients to demonstrate the validity of this approach.

## Methods

### Animals and study design

Male Lewis rats (2–3 months, *n* = 60, 270 ± 23 g) were purchased from Charles River (Sulzfeld, Germany). The rats were acclimated to the housing conditions for at least 5 days prior to any experimental procedures. Food and tap water were available ad libitum.

A focal delayed-type hypersensitivity model of experimental autoimmune encephalomyelitis (*f*DTH-EAE) was established as previously described [5, 15–17]. Briefly, an immune cell-mediated inflammatory response in bacillus Calmette–Guérin-induced intrastriatal lesions was triggered with complete Freund’s adjuvant, resulting in an active focal inflammatory lesion and a compromised blood–brain barrier during the acute phase of the inflammation (day 14). After 3 months (day 90), the lesions remodeled into well-defined chronic EAE lesions with repaired blood–brain barriers, which more accurately resembled chronic MS in humans [15, 16].

**Fig. 1:**
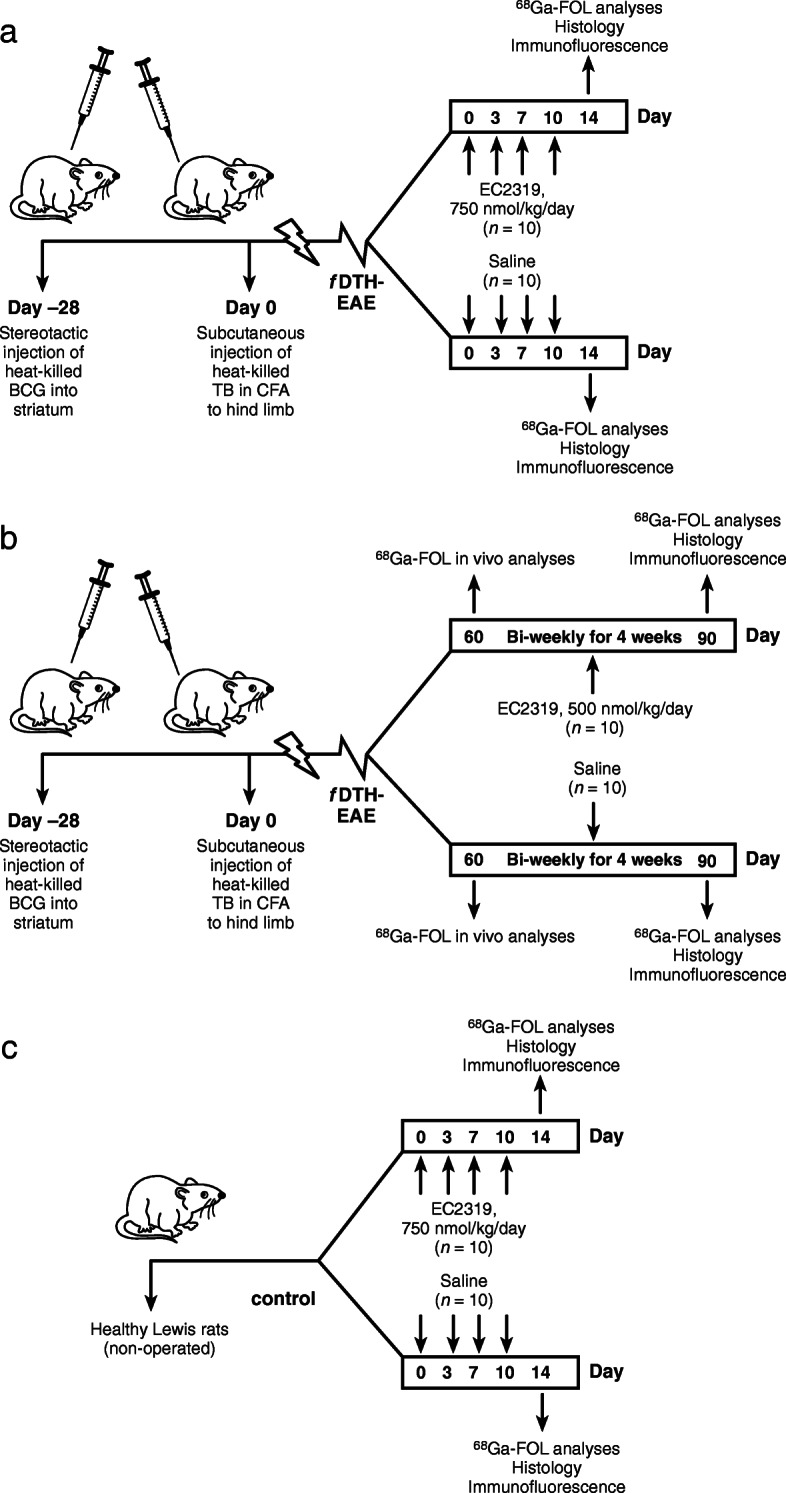
Study protocol for *f*DTH-EAE rats during acute and chronic phases and for healthy Lewis rats. **a** *f*DTH-EAE rats with acute inflammation were administered EC2319 (750 nmol/kg/day) or saline on days 0, 3, 7, and 10 after disease activation and were used for ^68^Ga-FOL, histology, and immunofluorescence analyses on day 14. **b** *f*DTH-EAE rats with chronic inflammation were administered EC2319 (500 nmol/kg/day) or saline biweekly for 4 weeks after the baseline in vivo evaluation 60 days after disease activation and were sacrificed for ^68^Ga-FOL, histology, and immunofluorescence analyses on day 90. **c** Healthy rats were administered EC2319 (750 nmol/kg/day) or saline on days 0, 3, 7, and 10 and sacrificed on day 14 for further analyses

The rats were randomly divided into 6 groups (*n* = 10/group) according to health status, duration of disease, and intervention (Fig. 1) and were put on a folate-free diet (5T0F:57W5; Testdiet, St. Louis, MO, USA) beginning 10 days before treatment. The rats in the acute phase (day 14) groups were subcutaneously administered with EC2319 (750 nmol/kg of body weight/day, 400 ± 100 μL in saline) in the nuchal area 0, 3, 7, and 10 days after lesion activation. The *f*DTH-EAE rats receiving only saline and healthy Lewis rats treated with EC2319 were used as controls. After the treatment, rats were imaged via PET/CT with ^68^Ga-FOL followed by sacrifice for ex vivo gamma counting of excised tissues. The brain cryosections were analyzed by autoradiography, histology, and immunofluorescence. The rats in the chronic phase (day 90) groups underwent ^68^Ga-FOL PET/CT at 60 days after lesion activation as a baseline measurement prior to the initiation of the biweekly treatment with subcutaneous EC2319 (500 nmol/kg/day, 520 ± 75 μL) for 4 weeks. PET/CT imaging was repeated at day 90, and the animals were sacrificed for ex vivo analyses as described above.

EC2319 is metabolically activated to release aminopterin and an aminopterin adduct. Plasma samples obtained from terminal blood samples were shipped frozen to Endocyte, Inc. and stored at − 80 °C until thawing for bioanalysis. EC2319, aminopterin, and the aminopterin adduct were extracted from the plasma (50 μL) by solid-phase extraction. The samples were then eluted into a 1.2-mL storage plate, evaporated, and reconstituted prior to ultra-performance liquid chromatography-tandem mass spectrometry. Incurred sample data were then generated by comparison with calibration curves from control plasma samples spiked for each compound.

### ^68^Ga-FOL studies

The precursor NOTA-folate was synthesized as previously described [13, 14]. ^68^Ga was obtained from a ^68^Ge/^68^Ga generator (Eckert & Ziegler Isotope Products, Burbank, CA, USA) by elution with 0.1 M HCl. ^68^Ga eluate (1.0 mL) was added to a mixture of 2-[4-(2-hydroxyethyl)piperazin-1-yl]ethanesulfonic acid (120 mg), gentisic acid (0.3 mg), and NOTA-folate (17 μg) in 120 μL TraceSELECT-grade water. The reaction mixture was heated at 80 °C for 10 min. After cooling to room temperature, the pH was adjusted to neutral with 1 M NaOH. The ^68^Ga-FOL was prepared in high radiochemical purity (> 95%) with the molar activity of 20–40 MBq/nmol at the end of synthesis. The duration of radiosynthesis was ~ 17 min.

In vivo imaging was performed with an Inveon multimodality small animal PET/CT device (Siemens Medical Solutions, Knoxville, TN, USA). The rats were anesthetized using a mixture of isoflurane and air (2.5%, 500 mL/min) on a heating pad, and a cannula was inserted into the tail vein. First, a 10-min CT was acquired for attenuation correction and anatomical reference. Then, ^68^Ga-FOL (45 ± 1 MBq, range 47–41 MBq) was injected IV for a 30-min PET acquisition as previously described [5].

The PET/CT data were reconstructed using a three-dimensional ordered-subset expectation maximization algorithm to time frames of 6 × 10 s, 3 × 20 s, 4 × 60 s, 3 × 180 s, and 3 × 300 s. Representative PET/CT images were captured with Inveon Research Workplace v.4.1, and the PET data were analyzed with Carimas v.2.9 (Turku PET Centre, Turku, Finland). Time frames of 17–30 min post-injection were used for quantitative PET image analysis. Spherical regions of interest were defined in the lesion hemisphere and mirrored onto the contralateral brain hemisphere, which served as an internal reference region. Quantitative PET image analysis was complemented by defining regions of interest to the heart left ventricle to obtain blood radioactivity concentrations. The results are expressed as standardized uptake value ratios [5].

Immediately after imaging, the animals were sacrificed and various tissues were excised, weighed, and measured for radioactivity with a gamma counter (Triathler 3′′; Hidex, Turku, Finland). The brains were frozen and cryosectioned for digital autoradiography [5]. A subset of brain cryosections was used to evaluate the in vitro binding of ^68^Ga-FOL [5]. The results are expressed as a percentage of the injected radioactivity dose per gram of tissue (%ID/g), organ/blood ratio, and bound-to-free ratio [5].

Analyses of in vivo stability and modeling of ^68^Ga-FOL PET data (Logan plots) were performed as previously described [5], with the exception that blood samples from healthy Lewis rats were withdrawn 5–30 min after ^68^Ga-FOL injection (*n* = 4 per time point).

### Histology and immunofluorescence

Paraformaldehyde-fixed 10-μm sections were stained with hematoxylin–eosin (H&E) and Luxol Fast Blue (LFB) with cresyl violet counterstain according to standard procedures. For double immunofluorescence staining, the sections were first fixed with ice-cold acetone for 3 min and washed with phosphate-buffered saline. Then, the sections were incubated for 30 min at room temperature with primary anti-human FR-β (which also recognizes rat FR-β; 1:50 dilution, m909; a kind gift from Professor Philip S. Low, Purdue University, West Lafayette, IN, USA) and anti-rat mannose receptor C type 1 (MRC-1) (1:2000 dilution; Abcam, Cambridge, UK), or with anti-rat inducible nitric oxide synthase (iNOS) (1:500 dilution; Abcam) antibodies for macrophages and microglia, or with anti-human FR-β and anti-rat CD68 antibodies (1:1000 dilution; AbD Serotec, Hercules, CA, USA) for macrophages/microglia. The sections were then incubated with fluorochrome-labeled secondary antibodies (1:100 dilution, anti-human Alexa Fluor 488 or anti-rat Alexa Fluor 594; Invitrogen of Thermo Fisher Scientific, Waltham, MA, USA) and counterstained with hematoxylin before mounting with ProLong Gold antifade reagent (P36930; Life Technologies of Thermo Fisher Scientific). The sections were scanned using a Pannoramic Midi fluorescence scanner (3D Histech, Budapest, Hungary) and analyzed with a Pannoramic viewer (3D Histech). The areas of positive staining or loss of LFB staining were determined from three to four brain sections and averaged for each rat. The amount of demyelination determined from the LFB staining was averaged to the total area of lesion hemisphere for each rat and expressed as area-%. The percentage of immunopositive FR-β signal from CD68-positive cells was determined from MS brain samples. The analysis was performed by using automatic thresholding of positively stained areas with the ImageJ v.1.48 software (National Institutes of Health, Bethesda, MD, USA). The iNOS/MRC-1 staining ratio was calculated for each rat. The lesion sizes were evaluated by manually defining regions of interest on the H&E-stained sections from each rat. Lymphocyte recruitment was quantified by determining lymphocyte count densities from three nonoverlapping areas within the lesion as observed with H&E staining. The areas were chosen according to the average density of recruited lymphocytes within the inflammatory lesion, and the results are expressed as lymphocyte count density per millimeter squared.

### Human tissue samples

Human formalin-fixed paraffin-embedded tissue samples (*n* = 5 MS brain samples; *n* = 5 normal brain samples) were obtained from Auria Biobank (Turku University Hospital, Turku, Finland). All samples had been taken at autopsy for histopathologic examination between 2001 and 2013. The samples presented homologous findings within both groups. For the CD68, iNOS, and MRC-1 double immunofluorescence staining, the samples were stained as described above. For immunohistochemistry, the sections were stained as previously described for anti-FR-β staining [5]. Positivity for FR-β immunohistochemistry is reported as negative, weak, moderate, or strong on the basis of the staining intensity.

### Cytokine and chemokine measurements in rat plasma

Plasma levels of interferon γ (IFN-γ) and interleukins IL-1β, IL-4, IL-6, and IL-10 were measured in duplicates with Luminex assay according to the manufacturer’s instructions (MILLIPLEX MAP Rat Cytokine/Chemokine Magnetic Bead Panel, Merck Millipore, MA, USA). The minimum detectable concentrations (pg/mL) for the analytes were 14.6 (IFN-γ), 12.2 (IL-1β), 4.9 (IL-4), 73.2 (IL-6), and 7.3 (IL-10).

### Statistical analysis

All statistical analyses were performed with the GraphPad Prism v.7.01 software (Graph Pad Software Inc., La Jolla, CA, USA). The results are presented as means ± SDs. Nonparametric Kruskal–Wallis tests with Mann–Whitney post hoc tests were used to compare tracer uptake values in lesion hemispheres between the groups as well as intragroup histological and immunofluorescence data. Spearman’s correlation was used to analyze the relationship between kinetic modeling and semiquantitative in vivo PET data. Two-way repeated-measures analysis of variance (ANOVA) was performed to compare cytokine levels between the groups. A result was considered statistically significant with a *P* value of < 0.05.

## Results

### EC2319 reduces FR-β expression, lesion size, and iNOS/MRC-1 ratio during chronic EAE

**Fig. 2:**
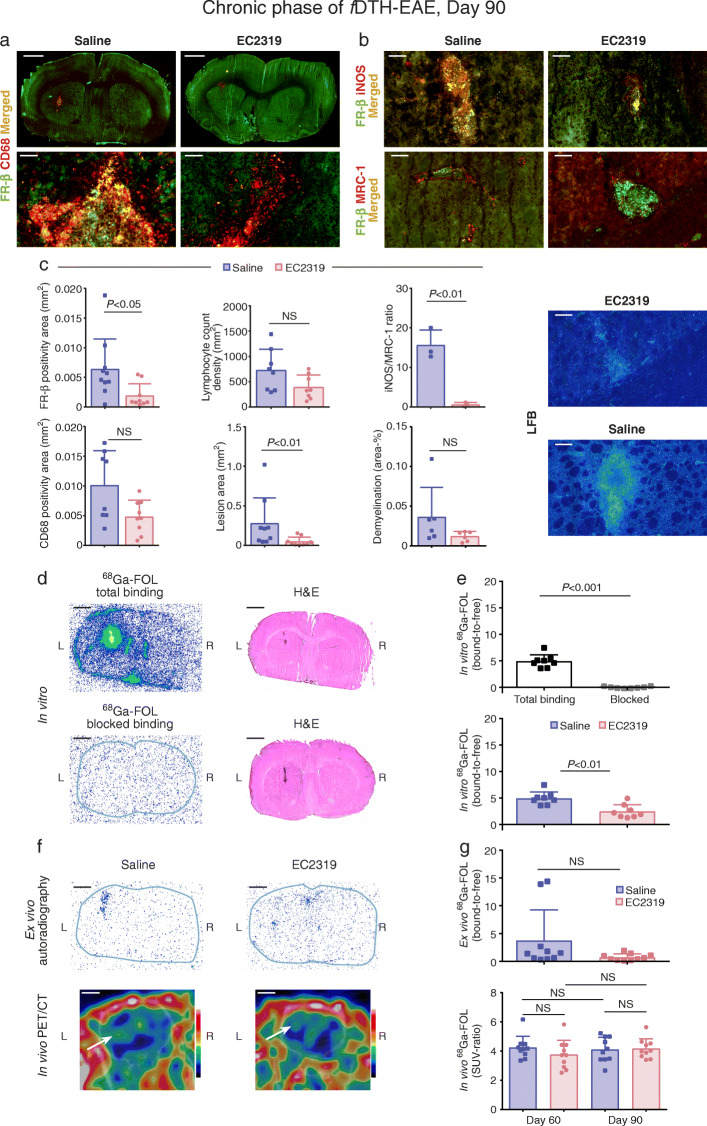
EC2319 treatment effects in rats during the chronic phase of *f*DTH-EAE. **a** Low- and high-power magnification photomicrographs of double immunofluorescence staining for FR-β and CD68 in *f*DTH-EAE rat brain cryosections. **b** High-power magnification photomicrographs of FR-β and iNOS or MRC-1 double staining in *f*DTH-EAE cryosections. Scale bars, 50 μm (high power) and 2 mm (low power). **c** Quantification of FR-β and CD68 positivity, lymphocyte count densities, lesion areas, iNOS/MRC-1 ratios, and demyelination areas on day 90 with representative LFB staining. **d** Representative in vitro autoradiographs of ^68^Ga-FOL total binding and folate glucosamine-blocked binding with corresponding H&E-stained sections. Scale bar, 2 mm. **e** Quantification of in vitro ^68^Ga-FOL total and folate glucosamine-blocked binding. **f** Representative ex vivo autoradiographs and in vivo PET/CT images of ^68^Ga-FOL from *f*DTH-EAE rats treated with EC2319 or saline. **g** Quantification of PET data at day 90. Error bars denote SDs. White arrows indicate lesion site

Biweekly EC2319 treatment was effective during the chronic phase of *f*DTH-EAE. Immunofluorescence staining revealed that FR-β expression was significantly reduced by 70% ± 10% in EC2319-treated rats compared with saline-treated controls (Fig. 2a, c; 0.0019 ± 0.00067 mm^2^ versus 0.0064 ± 0.0016 mm^2^, respectively, *P* = 0.017; *n* = 10/group). The area of CD68-positive cells was smaller following treatment but did not reach statistical significance (Fig. 2a, c; 0.010 ± 0.0021 mm^2^ [saline, *n* = 8] versus 0.0053 ± 0.0011 mm^2^ [EC2319, *n* = 9], *P* = 0.093). The demyelinated area assessed with LFB staining tended to be lower in EC2319-treated rats compared to saline-treated rats in the lesion hemisphere, but the difference did not reach statistical significance (Fig. 2c; 0.012% ± 0.0096% versus 0.036% ± 0.024%, respectively, *P* = 0.093; *n* = 6/group). The iNOS/MRC-1 ratio was significantly reduced by 96% ± 2% in EC2319-treated rats (Fig. 2b, c; 15.59 ± 2.24 [saline, *n* = 3] versus 0.63 ± 0.29 [EC2319, *n* = 3], *P* = 0.0027). Moreover, the lesion size was reduced in EC2319-treated *f*DTH-EAE rats during the chronic phase compared with that in the saline-treated group (Fig. 2c; 0.055 ± 0.018 [*n* = 9] versus 0.26 ± 0.098 [*n* = 10], respectively, *P* = 0.0056). At day 90, the chronic lesions had lower (but not statistically significant) lymphocyte count densities in EC2319-treated than in saline-treated rats (Fig. 2c; 393.30 ± 84.66 mm^2^ versus 729.80 ± 146.00 mm^2^, respectively, *P* = 0.065; *n* = 8/group).

**Fig. 3:**
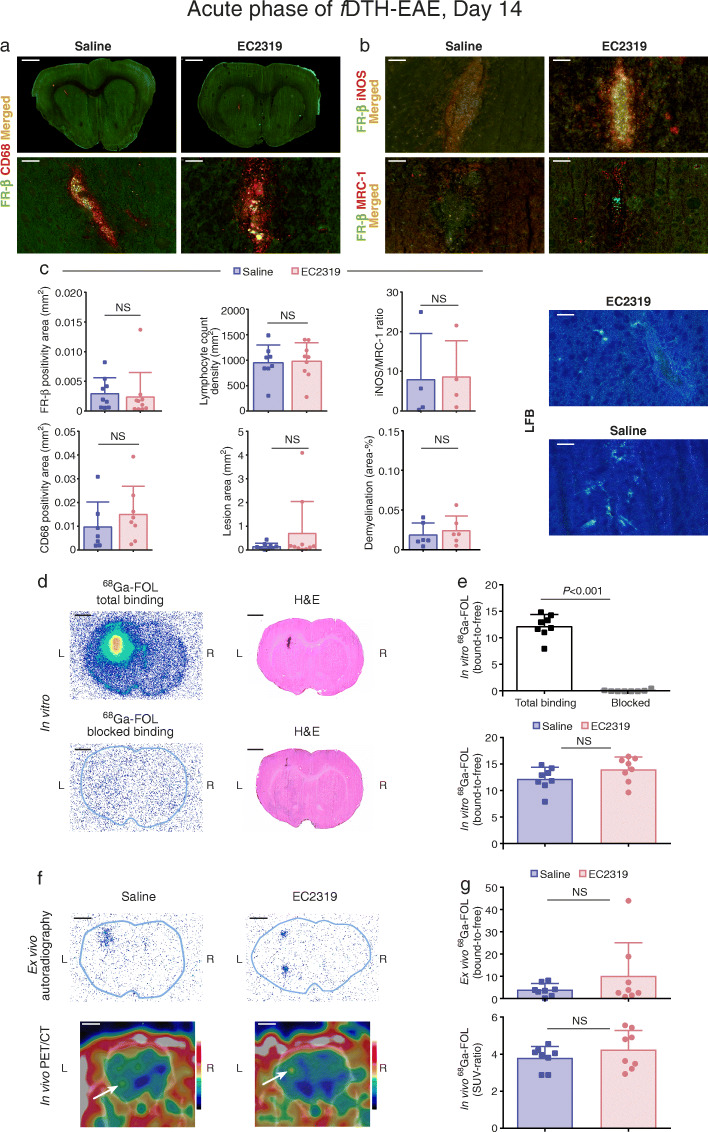
EC2319 treatment effects in rats during the acute phase of *f*DTH-EAE. **a** Low- and high-power magnification photomicrographs of double immunofluorescence staining for FR-β and CD68 in *f*DTH-EAE rat brain cryosections. **b** High-power magnification photomicrographs of FR-β and iNOS or MRC-1 double staining in *f*DTH-EAE cryosections. Scale bars, 50 μm (high power) and 2 mm (low power). **c** Quantification of FR-β and CD68 positivity, lymphocyte count densities, lesion areas, iNOS/MRC-1 ratios, and demyelination areas at day 14 with representative LFB staining. **d** Representative in vitro autoradiographs of ^68^Ga-FOL total binding and folate glucosamine-blocked binding with corresponding H&E-stained sections. Scale bar, 2 mm. **e** Quantification of in vitro ^68^Ga-FOL total and folate glucosamine-blocked binding. **f** Representative ex vivo autoradiographs and in vivo PET/CT images of ^68^Ga-FOL from *f*DTH-EAE rats treated with EC2319 or saline. **g** Quantification of PET data on day 14. Error bars denote SDs. White arrows indicate lesion site

In vitro ^68^Ga-FOL assays showed drastically lower binding when the brain cryosections were first incubated with the folate-glucosamine blocking agent than in those without any blocking agent, confirming that tracer binding is FR-specific (Fig. 2d, e). Tracer binding was significantly lower in the brain sections of EC2319-treated rats than in saline-treated rats (Fig. 2e; bound-to-free ratios, 2.47 ± 0.45 versus 4.93 ± 0.43, respectively, *P* = 0.0047; *n* = 8/group). ^68^Ga-FOL revealed the inflammatory lesions in in vivo PET/CT images and with ex vivo autoradiography*.* However, unlike the in vitro binding results, they did not show any therapeutic effect (Figs. 2f, g, 3f, g).

According to ex vivo gamma counting, the radioactivity concentrations were significantly higher in the blood and lower in lymph node and spleen tissues from EC2319-treated rats than those in saline-treated rats during the chronic phase of EAE (Table 1). The organ/blood ratios were markedly reduced in multiple organs, including the adrenal glands, heart, kidneys, liver, lungs, lymph node, muscle, pancreas, salivary glands, skin, skull, small intestine, spleen, and white adipose tissue from EC2319-treated rats compared with those from saline-treated rats (Table 2).

**Table 1:** Ex vivo biodistribution (percentage of injected dose per gram of tissue) of ^68^Ga-FOL at 30 min post-injection in rats with *f*DTH-EAE after treatment with EC2319 or saline

Organ	Day 14				Day 90			
	EC2319 (*n* = 8)	Saline (*n* = 8)	*P* value	Treatment effect	EC2319 (*n* = 10)	Saline (*n* = 10)	*P* value	Treatment effect
Adrenal glands	0.24 ± 0.04	0.25 ± 0.03	0.83	↓	0.16 ± 0.04	0.20 ± 0.05	0.05	↓
Blood	0.03 ± 0.01	0.014 ± 0.002	0.003	↑	0.06 ± 0.03	0.03 ± 0.03	0.03	↑
Brain	0.020 ± 0.004	0.011 ± 0.003	< 0.001	↑	0.02 ± 0.01	0.02 ± 0.01	0.46	↑
Heart	0.12 ± 0.02	0.19 ± 0.06	0.02	↓	0.09 ± 0.02	0.09 ± 0.02	0.95	↑
Kidneys	12.80 ± 2.02	6.71 ± 1.34	< 0.001	↑	8.21 ± 3.21	8.51 ± 1.99	0.81	↓
Liver	0.30 ± 0.05	0.47 ± 0.13	0.007	↓	0.22 ± 0.09	0.23 ± 0.06	0.74	↓
Lungs	0.14 ± 0.02	0.17 ± 0.05	0.11	↓	0.11 ± 0.02	0.12 ± 0.02	0.30	↓
Lymph node	1.00 ± 0.17	0.92 ± 0.13	0.30	↑	0.75 ± 0.22	0.95 ± 0.22	0.05	↓
Muscle	0.07 ± 0.01	0.09 ± 0.02	0.01	↓	0.05 ± 0.02	0.07 ± 0.02	0.09	↓
Pancreas	0.26 ± 0.04	0.38 ± 0.09	0.006	↓	0.20 ± 0.06	0.24 ± 0.06	0.26	↓
Plasma	0.05 ± 0.01	0.03 ± 0.02	0.05	↑	0.08 ± 0.03	0.06 ± 0.04	0.17	↑
Salivary gland	ND	ND	ND	ND	0.61 ± 0.33	0.82 ± 0.20	0.11	↓
Skin	0.26 ± 0.10	0.14 ± 0.05	0.02	↑	0.15 ± 0.04	0.17 ± 0.03	0.32	↓
Skull	0.08 ± 0.02	0.09 ± 0.02	0.48	↓	0.06 ± 0.03	0.06 ± 0.02	0.82	↓
Small intestine	0.44 ± 0.09	0.52 ± 0.11	0.1	↓	0.30 ± 0.08	0.37 ± 0.10	0.10	↓
Spleen	0.92 ± 0.32	1.52 ± 0.38	0.004	↓	0.56 ± 0.31	0.91 ± 0.39	0.04	↓
Urine	3.01 ± 2.69	2.35 ± 0.98	0.53	↑	7.22 ± 5.62	9.66 ± 12.70	0.59	↓
White adipose	0.05 ± 0.02	0.07 ± 0.02	0.04	↓	0.031 ± 0.01	0.035 ± 0.01	0.27	↓

Results are means ± SDs

*ND* not determined

**Table 2:** Ex vivo biodistribution (organ/blood ratio) of ^68^Ga-FOL at 30 min post-injection in rats with *f*DTH-EAE after treatment with EC2319 or saline

Organ	Day 14				Day 90			
	EC2319 (*n* = 8)	Saline (*n* = 8)	*P* value	Treatment effect	EC2319 (*n* = 10)	Saline (*n* = 10)	*P* value	Treatment effect
Adrenal glands	9.55 ± 4.32	17.55 ± 2.91	< 0.001	↓	3.02 ± 1.65	9.89 ± 6.92	0.01	↓
Brain	0.73 ± 0.16	0.80 ± 0.24	0.51	↓	0.48 ± 0.39	0.90 ± 0.58	0.08	↓
Heart	4.94 ± 2.68	13.72 ± 5.17	0.002	↓	1.70 ± 0.81	4.50 ± 3.35	0.03	↓
Kidneys	483.01 ± 135.74	480.04 ± 108.58	0.96	↑	168.51 ± 119.09	371.09 ± 188.35	0.01	↓
Liver	12.03 ± 5.98	34.06 ± 10.74	< 0.001	↓	4.49 ± 3.6	11.82 ± 8.8	0.03	↓
Lungs	5.57 ± 2.54	12.44 ± 3.99	0.0015	↓	2.14 ± 0.99	5.99 ± 4.21	0.02	↓
Lymph node	38.28 ± 13.38	64.84 ± 6.42	< 0.001	↓	14.83 ± 9.51	46.01 ± 30.30	0.01	↓
Muscle	2.82 ± 1.16	6.85 ± 1.97	< 0.001	↓	1.04 ± 0.58	3.47 ± 2.54	0.02	↓
Pancreas	10.19 ± 3.83	27.53 ± 7.49	< 0.001	↓	3.90 ± 2.24	11.94 ± 8.63	0.02	↓
Plasma	1.85 ± 0.78	2.17 ± 1.58	0.62	↓	1.43 ± 0.45	1.71 ± 0.11	0.08	↓
Salivary gland	ND	ND	ND	ND	11.98 ± 9.84	39.39 ± 24.62	0.01	↓
Skin	10.53 ± 8.71	10.30 ± 4.02	0.95	↑	2.47 ± 1.65	7.44 ± 4.34	0.01	↓
Skull	3.23 ± 1.58	6.32 ± 1.63	0.002	↓	1.19 ± 0.98	2.99 ± 2.28	0.04	↓
Small intestine	16.99 ± 7.46	37.82 ± 9.98	< 0.001	↓	5.71 ± 3.10	18.63 ± 14.02	0.02	↓
Spleen	36.02 ± 17.56	107.40 ± 24.24	< 0.001	↓	9.68 ± 3.79	47.82 ± 39.15	0.01	↓
Urine	102.82 ± 67.31	167.88 ± 76.67	0.09	↓	132.55 ± 92.40	218.37 ± 168.11	0.18	↓
White adipose	1.93 ± 0.81	5.07 ± 1.32	< 0.001	↓	0.61 ± 0.36	1.53 ± 0.88	0.01	↓

Results are expressed as mean ± SDs

*ND* not determined

### EC2319 is ineffective during the acute phase of focal EAE

During acute *f*DTH-EAE, no differences in FR-β or CD68 immunofluorescence were observed between the EC2319- and saline-treated groups (Fig. 3a, c). With four therapeutic doses of EC2319 (750 nmol/kg of body weight/day), there were also no differences in the iNOS/MRC-1 ratio, the density of lymphocytes, lesion size, or loss of LFB staining compared with those in saline-treated rats (Fig. 3b, c). In vitro ^68^Ga-FOL binding assays (Fig. 3d, e), in vivo PET/CT imaging, and ex vivo autoradiography (Fig. 3f, g) similarly revealed no differences between EC2319- and saline-treated rats. However, ex vivo gamma counting of excised tissues revealed lower radioactivity in the heart, liver, muscle, pancreas, spleen, and white adipose tissues from the EC2319-treated group but higher levels in the blood, brain, kidneys, plasma, and skin samples from rats receiving only saline (Table 1). The ^68^Ga-FOL organ/blood ratios were significantly lower in the adrenal glands, heart, liver, lungs, lymph node, muscle, pancreas, skull, small intestine, spleen, and white adipose tissue, indicating that EC2319 exerts a systemic anti-inflammatory effect (Table 2).

Ex vivo biodistribution results of ^68^Ga-FOL are presented in Table 3. In general, the biodistribution was similar to that reported previously [5]; the highest uptakes were seen in the urinary bladder and FR-positive kidneys. The results indicate that the administration of EC2319 systemically reduces the expression of FR-β and thus its availability for ^68^Ga-FOL, thereby augmenting the proportion of free ^68^Ga-FOL, which is detected in circulation, lipophilic tissues, and kidneys. This view is also supported by the organ/blood ratio data (Table 3).

**Table 3:** Ex vivo biodistribution of ^68^Ga-FOL at 30 min post-injection on day 14 in healthy Lewis rats

Organ	%ID/g				Organ/blood ratio			
	EC2319 (*n* = 10)	Saline (*n* = 10)	*P* value	Treatment effect	EC2319 (*n* = 8)	Saline (*n* = 8)	*P* value	Treatment effect
Adrenal glands	0.31 ± 0.09	0.35 ± 0.06	0.25	↓	5.92 ± 1.69	10.80 ± 2.19	< 0.001	↓
Blood	0.05 ± 0.02	0.03 ± 0.01	0.004	↑	ND	ND	ND	ND
Brain	0.04 ± 0.01	0.02 ± 0.003	< 0.001	↑	0.73 ± 0.13	0.59 ± 0.09	0.027	↑
Heart	0.18 ± 0.08	0.20 ± 0.03	0.37	↓	3.35 ± 1.51	6.19 ± 0.85	< 0.001	↓
Kidneys	13.18 ± 4.39	6.17 ± 1.13	< 0.001	↑	252.84 ± 84.26	170.46 ± 18.89	0.037	↑
Liver	0.26 ± 0.07	0.47 ± 0.15	0.03	↓	4.89 ± 1.31	14.92 ± 4.85	< 0.001	↓
Lungs	0.16 ± 0.03	0.22 ± 0.03	< 0.001	↓	3.03 ± 0.58	6.94 ± 0.79	< 0.001	↓
Lymph node	0.94 ± 0.25	0.88 ± 0.08	0.52	↑	17.96 ± 4.75	27.35 ± 2.01	< 0.001	↓
Muscle	0.11 ± 0.05	0.09 ± 0.01	0.12	↑	2.15 ± 0.89	2.66 ± 0.28	0.17	↓
Pancreas	0.29 ± 0.06	0.36 ± 0.04	0.16	↓	5.59 ± 1.05	9.96 ± 1.42	< 0.001	↓
Plasma	0.09 ± 0.03	0.06 ± 0.02	0.01	↑	1.73 ± 0.49	1.95 ± 0.61	0.89	↓
Salivary gland	1.01 ± 0.35	1.24 ± 0.21	0.10	↓	19.28 ± 6.74	38.39 ± 6.48	< 0.001	↓
Skin	0.23 ± 0.07	0.21 ± 0.08	0.69	↑	4.33 ± 1.32	5.99 ± 2.08	0.04	↓
Skull	0.07 ± 0.03	0.15 ± 0.04	< 0.001	↓	1.32 ± 0.54	4.81 ± 1.16	< 0.001	↓
Small intestine	0.56 ± 0.15	0.78 ± 0.10	0.002	↓	10.70 ± 2.87	24.30 ± 2.49	< 0.001	↓
Spleen	0.75 ± 0.31	1.83 ± 0.37	< 0.001	↓	14.45 ± 5.88	55.95 ± 11.99	< 0.001	↓
Urine	3.00 ± 2.08	2.86 ± 0.02	0.32	↑	57.62 ± 39.86	92.25 ± 39.95	0.13	↓
White adipose	0.05 ± 0.02	0.03 ± 0.01	0.004	↑	1.04 ± 0.17	1.45 ± 0.55	0.04	↓

Results are expressed as mean ± SDs

*ND* not determined

The plasma cytokine measurements from *f*DTH-EAE and healthy Lewis rats revealed no significant differences in IFN-γ, IL-4, and IL-6 concentrations between the EC2319- and saline-treated groups (Table 4). However, EC2319 treatment of healthy Lewis rats reduced the levels of IL-1β (*P* = 0.02) and IL-10 (*P* = 0.03). In addition, there were differences in baseline levels (in saline-treated groups): plasma concentrations of IFN-γ, IL-1β, IL-4, and IL-10 were significantly lower in chronic *f*DTH-EAE versus healthy rats (*P* < 0.05), and IL-6 was significantly lower in acute *f*DTH-EAE versus healthy rats (*P* < 0.05).

**Table 4:** Rat plasma levels of cytokines (pg/mL)

Cytokine	*f*DTH-EAE day 14			*f*DTH-EAE day 90			Healthy Lewis rats		
	EC2319 (*n* = 2)	Saline (*n* = 4–6)	*P* value	EC2319 (*n* = 2–4)	Saline (*n* = 5–7)	*P* value	EC2319 (*n* = 4–6)	Saline (*n* = 3–7)	*P* value
IFN-γ	ND	202 ± 152	0.92	216 ± 184	95 ± 77^b^	0.29	486 ± 507	558 ± 755^b^	0.81
IL-1β	662 ± 614	441 ± 284	0.53	205 ± 178	203 ± 79^b^	0.97	321 ± 167	562 ± 316^b^	0.02
IL-4	ND	42 ± 29	0.60	28 ± 17	29 ± 22^b^	0.96	70 ± 70	234 ± 237^b^	0.09
IL-6	ND	442 ± 266^a^	0.74	ND	ND	ND	486 ± 386	5210 ± 4146^a^	0.06
IL-10	780 ± 548	604 ± 290	0.58	417 ± 259	395 ± 121^b^	0.82	479 ± 194	700 ± 297^b^	0.03

Results are means ± SDs. *P* values are from two-way repeated-measures ANOVA

*ND* not determined

^a^*f*DTH-EAE day 14 versus healthy Lewis rats *P* < 0.05

^b^*f*DTH-EAE day 90 versus healthy Lewis rats *P* < 0.05

### FR-β expression is increased in MS brain samples

**Fig. 4:**
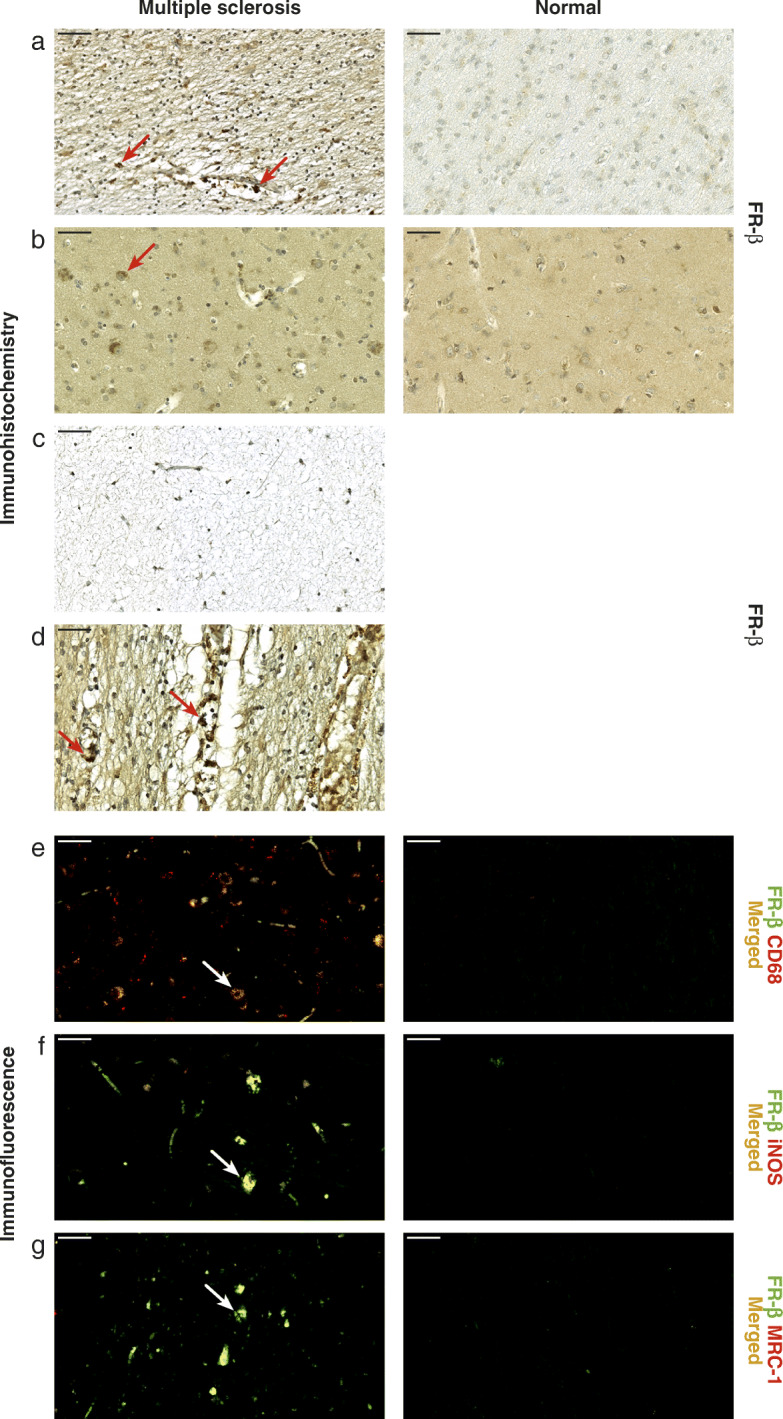
Immunohistochemistry with postmortem sections from MS and normal human brains. **a** Immunohistochemistry reveals FR-β expression in normal-appearing white matter from a patient with secondary progressive MS (left) but not in the white matter from a normal brain (right). **b** Moderate FR-β expression is observed in the normal-appearing gray matter from a patient with secondary progressive MS (left) but not in a normal brain (right). **c** Chronic inactive lesions display minimal or no FR-β expression. **d** FR-β-immunoreactive macrophages border chronic active plaques. Immunofluorescence staining reveals that FR-β colocalizes with CD68 (**e**), iNOS (**f**), and MRC-1 (**g**) in normal-appearing white matter from a patient with secondary progressive MS (left) but not in the white matter of a normal brain (right). Scale bars, 50 μm. Red arrows indicate FR-β positivity, and white arrows indicate colocalization of FR-β and CD68, iNOS, or MRC-1

To demonstrate the translational relevance of these findings, we assessed the expression of FR-β using postmortem brain sections from MS patients and controls. FR-β was moderately expressed in normal-appearing white matter but weakly expressed or absent in normal-appearing gray matter and in chronically inactive white matter plaques compared with normal human brain tissues of corresponding anatomical areas (Fig. 4a–c). In addition, the chronic active lesions displayed moderate levels of FR-β at the border of chronic lesions (Fig. 4d), typically in areas known to exhibit macrophages [18]. Double immunofluorescence staining revealed that FR-β colocalized with CD68, iNOS, and MRC-1 in MS brain tissue samples but not in normal brain (Fig. 4e–g), and occasionally, the blood vessels at the lesion sites showed immunopositivity for FR-β. Quantification revealed that 62.7% ± 13.0% (*n* = 5) of CD68-positive cells in MS brain lesions were FR-β-positive.

### EC2319 was well tolerated in rats

**Fig. 5:**
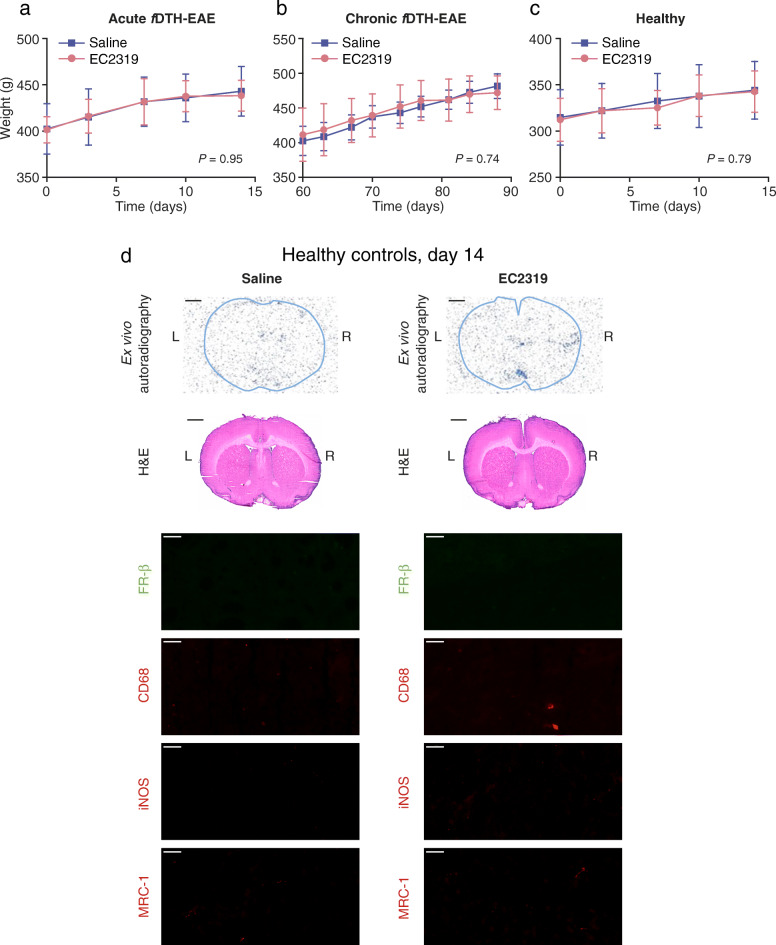
Body weights, digital autoradiography, histology, and immunofluorescence of healthy rats. The body weights of *f*DTH-EAE rats during acute (**a**) and chronic (**b**) phases of the disease and of healthy Lewis rats (**c**) with EC2319 or saline treatment. **d** Ex vivo ^68^Ga-FOL autoradiography, H&E histology, FR-β, CD68, iNOS, and MRC-1 immunofluorescence staining. Scale bars, 50 μm (high power) and 2 mm (low power)

EC2319 treatment was safe and well tolerated by the rats, with no effect on body weight in any of the studied groups (Fig. 5a–c). At the time of the PET studies, the plasma levels of EC2319 and its active metabolites were under the detection limits of ultra-performance liquid chromatography-tandem mass spectrometry (the calibrated ranges were 3.0–600 ng/mL for EC2319 and 0.3–120 ng/mL for both aminopterin and the aminopterin adduct; data not shown). In addition, healthy rats treated with EC2319 or saline showed no lesions; no immunopositivity for FR-β, CD68, iNOS, or MRC-1; and no uptake of ^68^Ga-FOL in the brain (Fig. 5d).

## Discussion

In this work, we evaluated the efficacy of the novel folate-aminopterin conjugate EC2319 for the treatment of acute and chronic *f*DTH-EAE, a rat model of MS. *f*DTH-EAE is a clinically relevant model for assessing lesion characteristics, immune cell populations, and therapy responses [16, 17]. We report here, for the first time, that EC2319 effectively reduces lesion size, FR-β expression, iNOS/MRC-1 ratio, and ^68^Ga-FOL binding in vitro during the chronic phase of neuroinflammation, although the effects were not apparent during the acute phase. Most intriguing, however, we found that FR-β is expressed in the brain lesions of patients with MS.

In line with previous studies on inflammatory autoimmune diseases [19, 20], we demonstrated that the FR-β expressed in EAE lesions colocalized with iNOS, which is expressed by pro-inflammatory macrophages/microglia during acute and chronic phases of inflammation. Although EC2319 did not alter the number of iNOS-positive cells, it did restore the iNOS/MRC-1 equilibrium in rats with *f*DTH-EAE. Similar effects were reported in EAE models treated with flavocoxid, fasudil, or exosomes from bone marrow mesenchymal stem cells, but these agents have not been tested in clinical trials [21–23]. The shift in macrophage/microglia polarization toward the iNOS-positive (M1) and away from the MRC-1-positive (M2) phenotype in relapsing EAE is known to predict inflammation severity [24]; thus, restoring the equilibrium between M1- and M2-type cells is important for recovery [24, 25]. Our data suggests that FR-β is expressed only in a certain subpopulation of CD68-positive cells in *f*DTH-EAE. As observed during the chronic phase of inflammation, most of the FR-β immunopositive signal appears to originate from iNOS-positive cells in the lesion (Fig. 2b). Based on this finding, EC2319 is therefore most likely targeting iNOS-positive pro-inflammatory cells rather than MRC-1-positive cells. This supports the view that EC2319 can help to regulate the inflammatory processes in the CNS that are impaired in acute and chronic EAE [26] and also in MS [27].

EC2319 represents the best-in-class folate-aminopterin conjugate with a similar mechanism of action as EC0746, the first compound of this class, differing only in the linker design [8]. In a FR-dependent manner, EC2319 induces cell cycle arrest (anti-proliferation), modulates inflammatory cytokine/chemokine responses, and demonstrates both local and systemic anti-inflammatory response. In addition, EC2319 shuts down a subset of inflammatory monocytes in multiple disease models (all part of a separate manuscript that is currently under review). Here in our study, EC2319 reduced the lesion size in the *f*DTH-EAE rat model. This consequently appeared to cause the reduction of FR-β immunopositive signal and the restoration of iNOS/MRC-1 equilibrium. FR-β is largely absent from other cells known to infiltrate the CNS in the EAE rat model, such as T lymphocytes [28]. This data suggest that the likely targets of EC2319 are the inflammatory CD68-positive cells, which infiltrate the CNS during the active phase of inflammation. In addition, these cells expressing functional FR-β are present in both CNS and peripheral sites of inflammation [28], and thus, the suppression of peripheral immune cells following EC2319 treatment may further explain the reduced lesion size observed in the chronic phase of *f*DTH-EAE.

Folate-conjugated therapies, such as FR-mediated antifolates or FR-targeted immunotherapies, have shown efficacy for the treatment of inflammatory conditions, including early rheumatoid arthritis in animal models [7]. However, there is very little information about their efficacy in chronic inflammatory conditions [7, 8]. Our results suggest that EC2319 may be an effective therapy for patients with a chronic progressive form of MS, for which there are very few effective therapies [2]. This finding may potentially expand the therapeutic indications for folate-aminopterin therapies that were initially limited to the treatment of acute peripheral inflammatory disorders. We show that the lesions in human tissue samples from patients with chronic progressive MS have FR-β-positive cells similarly to the lesions in the *f*DTH-EAE animal model suggesting the translational potential of these findings. Brain samples from MS patients were obtained from late-stage chronic lesions known to have significantly lower numbers of CD68-positive cells than in acute MS lesions [29]. This explains why we detected only a few FR-β-positive cells in the lesions. Based on our data, however, the majority of these CD68-positive cells expressed FR-β. In addition, we observed occasional FR-β positivity in lesion vasculature that could possibly be due to CD68-positive macrophages surrounding or entering the blood vessels. It is noteworthy that although BCG-induced chronic DTH lesions closely resemble those observed in the progressive phase of MS [15], there is little evidence that this EAE model could mimic chronic inactive lesions typically present in late stages of progressive MS limiting the extrapolation of these findings to humans. Most importantly, as EC2319 treatment reduced FR-β-positive cells in the focal EAE lesions, further studies are needed to determine if this would affect the long-term efficacy and safety of folate-aminopterin therapies, which rely on FR-mediated endocytosis of the anti-inflammatory drug conjugate. In addition, EC2319 therapy had no significant effect on cytokine levels in plasma samples collected in both acute and chronic phases of *f*DTH-EAE suggesting a mild systemic inflammation at the end of the study in this model. Interestingly, both pro-inflammatory cytokines (INF-γ, IL-1β) and anti-inflammatory cytokines (IL-4, IL-10) were significantly lower in *f*DTH-EAE rats during chronic inflammation (saline-treated) compared to healthy Lewis rats. This may refer to impaired immunoregulation during chronic inflammation in *f*DTH-EAE, which may contribute to triggering and sustaining inflammation. Although the mechanisms of action were beyond the scope of the current study, we recognize that brain cytokine levels may be more informative regarding the mode of action than the plasma levels measured in this work.

It is not clear why EC2319 was not effective in the acute phase of *f*DTH-EAE. Another folic acid-conjugated aminopterin analog, EC0746, was highly effective in the acute phase of myelin basic protein-induced EAE, improving disease-related scores and reducing inflammation and demyelination [4]. However, the discrepancy may reflect the inherent differences in the disease models. In rats, myelin basic protein-induced EAE induces a severe and acute disseminated inflammatory response throughout the brain and spinal cord with overt blood–brain barrier breakdown [4, 26], whereas *f*DTH-EAE initially induces small focal inflammatory lesions before progressing to a chronic stage with more diffuse inflammation and widespread macrophage/microglia activation [16]. Therefore, the relative expression of FR-β on CD68-positive cells during the acute phase of inflammation is likely lower in the *f*DTH-EAE brain, limiting the anti-inflammatory efficacy of EC2319. As the inflammation progresses toward the chronic phase, the increase in FR-β expression facilitates EC2319 binding and activity. Regardless of the phase of inflammation, however, *f*DTH-EAE exhibits only a focal lesion and hence very mild clinical symptoms during acute and chronic phases of the disease limiting the capability to investigate EC2319 therapy effects in reducing the clinical severity of the disease in *f*DTH-EAE [15, 16]. The observed therapeutic effect during the chronic phase may also be partly attributable to nontargeted effects of EC2319 metabolites. However, like EC0746, EC2319 demonstrates FR-specific activity in vitro, as well as in animal models of adjuvant arthritis, anti-glomerular basement membrane glomerulonephritis, and experimental autoimmune uveitis (data not shown). The apparent alteration of ^68^Ga-FOL uptake in normal tissues of both healthy rats and those with *f*DTH-EAE may be an artifact of the folate-deficient diet and FR competition following EC2319 treatment. However, EC2319 is also likely to have a systemic effect via FR-positive macrophages outside CNS that typically respond to antifolate therapy.

As a crude measurement of gross toxicity, the absence of any effect of EC2319 on animal body weight suggests that it was safe and well-tolerated. However, some of the rats demonstrated enlarged spleens with vesicles irrespective of their health status, disease duration, or intervention. This might have been a result of the folate-deficient diet used during the experimental protocol, as folate (B9 vitamin) deficiency can lead to mild-to-moderate megaloblastic anemia, wherein macrocytic erythrocytes are sequestered in the spleen as multiple small splenic lesions [30].

## Conclusions

The results presented here demonstrate FR-β expression in lesions in rats with *f*DTH-EAE and in patients with MS. EC2319, a folate-aminopterin drug conjugate, appears to be safe for use during acute and chronic *f*DTH-EAE. EC2319 effectively attenuated the inflammation and lesion burden in rats with chronic EAE, but not during the acute phase of inflammation. Although short-term treatment with EC2319 demonstrated beneficial effects in chronic EAE lesions, its long-term efficacy and safety remain to be determined. For the first time, we show that the MS patients have FR-β-positive cells in chronic active plaques, which indicates the translational relevance of these findings.

## Data Availability

Data supporting the conclusions of this article are presented in the manuscript.

## Abbreviations

%ID/g: Percentage of injected radioactivity dose per gram of tissue
^68^Ga-FOL: ^68^Ga-labeled 1,4,7-triazacyclononane-1,4,7-triacetic acid-conjugated folate
CD68: Cluster of differentiation 68
EAE: Experimental autoimmune encephalomyelitis
EC2319: Folate-conjugated aminopterin
*f*DTH-EAE: Focal delayed-type hypersensitivity model of experimental autoimmune encephalomyelitis
FR-β: Folate receptor-β
H&E: Hematoxylin–eosin
iNOS: Inducible nitric oxide synthase
MRC-1: Mannose receptor C type 1
MS: Multiple sclerosis
